# Three-Dimensionally Printed Elastic Cardiovascular Phantoms for Carotid Angioplasty Training and Personalized Healthcare

**DOI:** 10.3390/jcm13175115

**Published:** 2024-08-28

**Authors:** Krystian Jędrzejczak, Arkadiusz Antonowicz, Beata Butruk-Raszeja, Wojciech Orciuch, Krzysztof Wojtas, Piotr Piasecki, Jerzy Narloch, Marek Wierzbicki, Łukasz Makowski

**Affiliations:** 1 Faculty of Chemical and Process Engineering, Warsaw University of Technology, Waryńskiego 1, 00-645 Warsaw, Poland; 2 Eurotek International Sp.z o.o., Skrzetuskiego 6, 02-726 Warsaw, Poland; 3 Interventional Radiology Department, Military Institute of Medicine-National Research Institute, Szaserów 128, 04-141 Warsaw, Poland

**Keywords:** 3D printing, atherosclerosis, carotid artery stenting, percutaneous angioplasty, PIV

## Abstract

**Background/Objective:** Atherosclerosis is becoming increasingly common in modern society. Owing to the increasing number of complex angioplasty procedures, there is an increasing need for training in cases where the risk of periprocedural complications is high. **Methods:** A procedure was developed to obtain three-dimensional (3D) models and printing of blood vessels. The mechanical and optical properties of the printed materials were also examined. Angioplasty and stent implantation were tested, and the phantom was compared with the clinical data of patients who underwent interventional treatment. Both laser techniques and cone-beam computed tomography of the phantoms were used for comparison. **Results:** The printed material exhibited mechanical parameters similar to those of blood vessel walls. The refractive index of 1.473 ± 0.002 and high transparency allowed for non-invasive laser examination of the interior of the print. The printed models behaved similarly to human arteries in vivo, allowing training in treatment procedures and considering vessel deformation during the procedure. Models with stents can be analyzed using laser and cone-beam computed tomography to compare stents from different manufacturers. **Conclusions:** The developed methodology allows for simple and time-efficient production of personalized vessel phantoms.

## 1. Introduction

Carotid atherosclerosis is becoming increasingly common in modern society. Percutaneous angioplasty and carotid artery stenting are widely used alternatives to carotid endarterectomy for symptomatic and asymptomatic patients with hemodynamically significant carotid stenosis [1,2]. Due to the increasing number of patients with complex carotid artery atherosclerotic plaques and tortuous vascular anatomy, there is a need to ensure the best possible medical procedure outcomes and safety. Three-dimensional (3D) printing is becoming increasingly popular for planning patient-specific medical treatments [3]. The development of medical diagnostic imaging methods allows us to obtain sophisticated and detailed blood vessel models for better planning of endovascular procedures, simulation of physiological or pathological conditions, teaching of health care professionals, and outcome prediction. Three-dimensional blood vessel models can be created using computed tomography (CT) [4,5,6,7,8,9,10], magnetic resonance imaging (MRI) [10,11,12,13,14,15,16], optical coherence tomography (OCT) [17,18,19,20,21], near-infrared spectroscopy [22], and ultrasound imaging [23,24,25,26]. These models can be used for the numerical analysis of blood flow in vessels for training purposes and to plan the safest path during the procedure. Particularly, there is a high risk of serious periprocedural complications, i.e., severe vasospasm, vessel injury, or embolism. The development of 3D printing has allowed the design of accurate blood vessel models and mechanical parameters similar to those of living tissues. This allowed us to test different medical devices in vitro, such as carotid stents, without using human or animal tissues. This study describes the full path of developing training models of carotid arteries with atherosclerotic stenosis and complicated atherosclerotic plaques—“unstable plaques”. This allowed us to assess the mechanical parameters of the stents and to test various stent implantation scenarios.

Moreover, apart from being used as training material, a 3D-printed elastic artery can be used to experimentally measure fluid flow in an artery while measuring pressures analogously to constant Resistance Ratio (cRR) or Fractional Flow Ratio (FFR) measurements. A system was developed to test the possibility of micro Particle Image Velocimetry (µPIV) measurements in flexible artery prints and to measure geometry deformation after stenting or angioplasty. Measurements of geometric deformation were compared with the results of the CT scans of the stented 3D models. Moreover, the 3D-printed flexible model can help modify the stent grafts [27,28,29] due to its versatility and mechanical properties similar to arterial walls.

## 2. Materials and Methods

### 2.1. Geometry Preparation

Geometries were obtained using carotid CT angiography. The DICOM files were used to create 3D models of arteries with atherosclerotic plaques and aneurysms. Subsequently, the models were exported as .stl files. Subsequently, the raw 3D models were edited using Autodesk Meshmixer 3.5 to eliminate distortions observed in the models as small spikes resulting from the finite resolution of clinical imaging. The preliminary denoised 3D models were exported to ANSYS SpaceClaim 2023R2 in the next stage. In this software, the shrinkwrap tool was used to remove spare noise from the models. The complexity of the facets was reduced using Auto Skin. Next, the geometries were trimmed at the ends. The obtained geometries were the fluid volumes. The raw model was copied and edited using sculpting tools to reconstruct its shape without constriction to capture the physiological thickness of the arteries in the stenotic region. These models were also revised in ANSYS SpaceClaim, similar to the previous models. However, the Shell tool was used after Auto Skin to create a 0.75 mm thicker model. The cleaning procedure was repeated. Both models were exported to the ANSYS DesignModeler 2023R2. In ANSYS DesignModeler, Boolean operations were used to create a hollow shell geometry and separate the fluid volume. The obtained geometries were subsequently extruded for better 3D printing and connected to the experimental setup.

### 2.2. Three-Dimensional Printing

**Figure 1:**
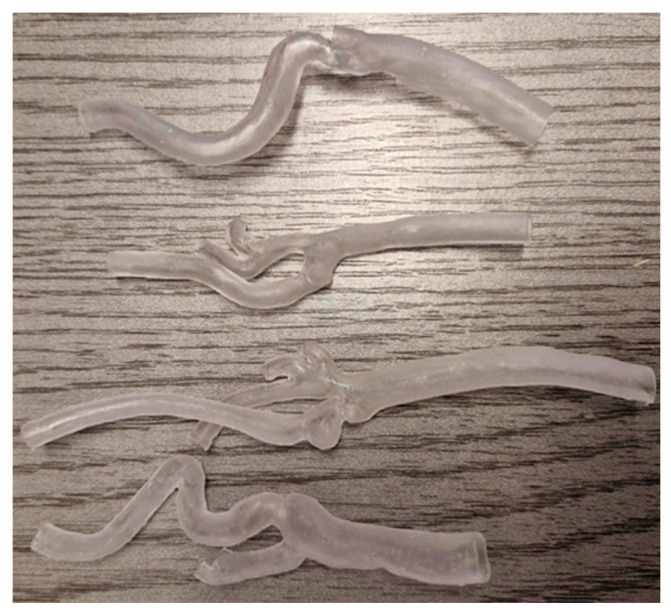
Three-dimensionally printed geometries after post-processing.

The geometries of the arteries were 3D-printed using Form 3B+ (Formlabs, Somerville, MA, USA). The BioMed Elastic 50A V1 resin (Formlabs, Somerville, MA, USA) can mimic mechanical properties and ensure biocompatibility. This resin is a translucent medical-grade material with elastic properties. Three-dimensional models were printed with 100 µm layer thickness, as recommended by the manufacturer. Build Platform 2 ensured biocompatibility because of the stainless-steel printed surface and quick-release technology. After 3D printing, the printouts were washed for 20 min in 99% isopropyl alcohol (IPA) using Form Wash (Formlabs, Somerville, MA, USA). The dried-out printouts were cured using a Form Cure L (Formlabs, Somerville, MA, USA). The printouts were placed in glass beakers and submerged in water during curing. The curing temperature was set to 70 °C for 30 min with Ultraviolet (UV) light and 5 min pre-heat without UV light. After curing, the supports were removed, and the remaining pieces were polished using Finishing Tools from Formlabs (Somerville, MA, USA). The obtained models are shown in Figure 1.

### 2.3. Mechanical and Optical Parameters Measurements

Artery walls have elastic properties; the Young’s moduli of healthy and atherosclerotic tissues are 1.5 MPa [30] and 3.8 MPa [31], respectively. Simultaneously, the density of the arterial wall varied from 1120 kg/m^3^ [32] for healthy tissue to 1220 kg/m^3^ [33] for atherosclerotic tissue. Cylindrical samples with an internal diameter of 5 mm and wall thickness of 1 mm (n = 6 for each type) were placed in the pneumatic jaws of a testing machine (Instron 3345, Norwood, MA, USA) equipped with a 50 kN static load cell. The samples were stretched at a rate of 5 mm/min until they broke. Young’s modulus (YM) was automatically calculated from the stress–strain curve using Bluehill 3 software. The results were presented as a mean value ± SD. The statistical significance of the differences was analyzed using a single-factor analysis of variance (ANOVA) for *p* < 0.05, with a post hoc Tukey’s test (software OriginPRO 8.0, OriginLab Corporation, Northampton, MA, USA). 

**Figure 2:**
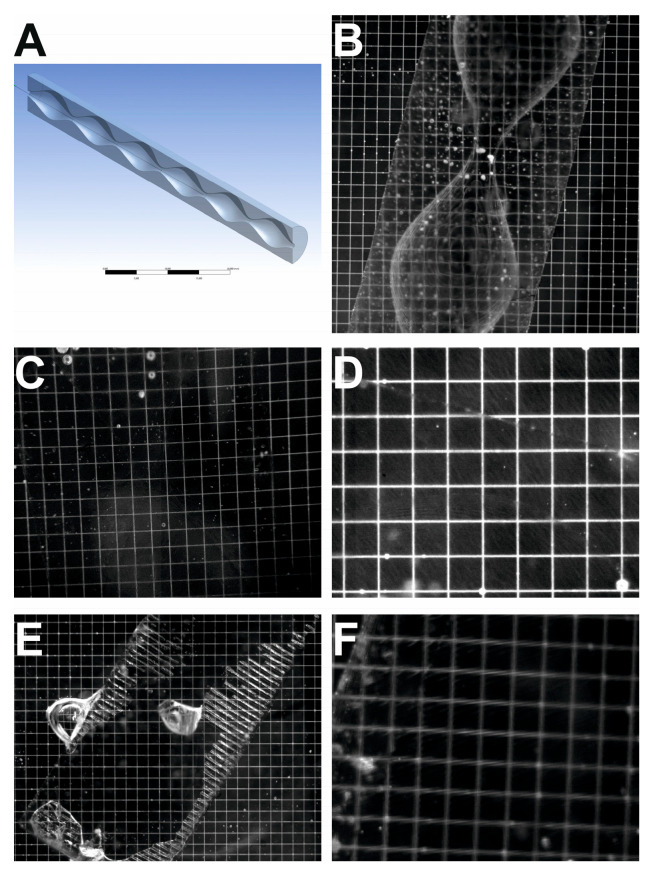
(**A**) Geometry used for refractive index matching and printing accuracy measurements. (**B**) Refractive index matching for glycerin solution without NaI; RI = 1.3965. (**C**) Refractive index matching for glycerin solution with NaI; RI = 1.4741. (**D**) Refractive index matching for glycerin solution with NaI; RI = 1.4730. (**E**) Refractive index matching for glycerin solution with NaI; RI = 1.4716. Almost invisible stenosis shape. (**F**) Refractive index matching for glycerin solution with NaI; RI = 1.4875.

The densities and refractive indices of the printed blocks were also measured. The blocks were weighed, and the density was determined based on the measured volume. The refractive index was measured using an Abbe refractometer, and the comparative method involved selecting a solution with a refractive index at which there was no refraction of light at the liquid–solid interface. A typical direct measurement of a printed object using an Abbe refractometer provided a weak and blurred partition boundary. Refractive index matching was conducted using a 3D print with half of the multiple stenosis vessel model, with a 1 mm diameter for the stenosis and a 5 mm diameter at its widest point, as shown in Figure 2A. The testing geometry was used alongside a calibration plate with a 0.5 mm grid. The system with the glycerin solution, shown in Figure 2B, was used as an example of clear light refraction at the interface and to assess the print accuracy owing to the calibration plate. To determine the exact value of the refractive index (RI), a series of tests were performed for 12 liquids with different refractive indices in the range of 1.464–1.485. Figure 2C,E show a system in which light refraction is almost imperceptible, proving that the correct refractive index is close to the refractive index of these solutions. A dark contour in the shape of a single hourglass can barely be seen in Figure 2C, tilted slightly to the left, with small air bubbles on the top. The same shape tilted to the right with an air bubble during the narrowing of the geometry can be observed in Figure 2E because only the transverse strips contain air. Figure 2D shows the almost invisible edge of the bulge of geometry at the bottom of the image and a straight diagonal line on the top of the image, which represents the outer edge of the 3D print. Figure 2F shows deformation when the RI deviates approximately 0.01 from the correct value.

**Figure 3:**
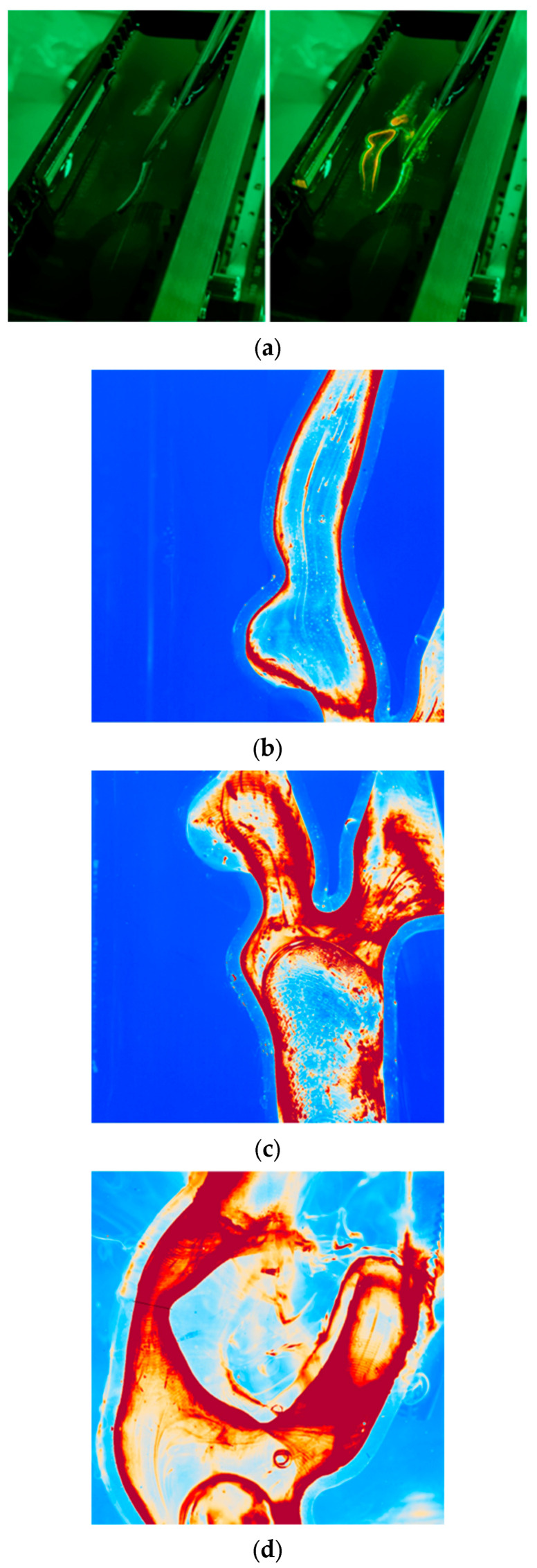
(**a**) Experimental setup for measurements of internal dimensions of 3D prints, (left) laser off and (right) laser on. (**b**) PIV laser-illuminated geometry contours. (**c**) PIV laser-illuminated complex geometry contours. (**d**) Marking the line of comparison dimension between the 3D print and virtual model of stenosis.

Obtaining the value of the refractive index allowed the development of a system for optical analysis of geometric dimensions. The system consisted of a cuboidal pool filled with glycerin and the geometry of an artery connected to internal spigots to which, on the other side, flexible tubing was connected to flood the system with a mixture of glycerin, water, and sodium iodide stabilized with sodium thiosulfate with the addition of rhodamine as a fluorescent dye. The selection of an appropriate refractive index ensured no light refraction, which made the geometry of the artery invisible, as shown in Figure 3a on the left. However, the inside of the artery was visible when the laser was operating, as shown on the right side of Figure 3a. The system consisted of a lens with a camera that recorded subsequent exposures. Figure 3b–d show images acquired by the camera from the same region as that in Figure 3a. The red colors originated from rhodamine deposition on the 3D-printed geometry inner walls induced by a 532 nm pulsed laser. The light blue halo represents the geometric walls.

### 2.4. Experimental Setup

**Figure 4:**
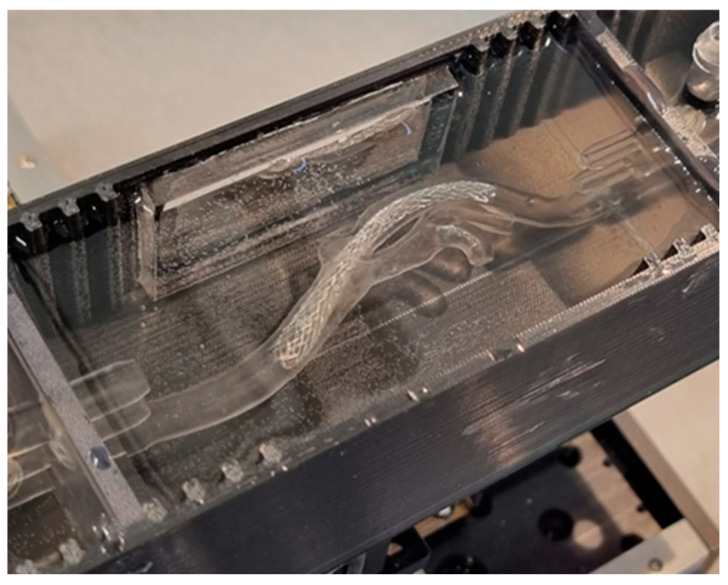
Experimental setup for percutaneous carotid artery stenting procedure.

An experimental system was prepared for live observation and video recording for subsequent analyses to test the possibility of training the percutaneous carotid artery stenting procedure. The 3D prints were fixed at the two ends by connectors with stubs, the rigid connectors were fixed with pliers, and the pliers were rigidly mounted on a tripod. The printout consisted of a camera with a lens that captured time-lapse photographs at a frequency of 7 Hz. The experimental setup for the test procedure is shown in Figure 4.

Images from the experiment were analyzed to determine the geometric deformation, with particular emphasis on arterial dilation in atherosclerotic stenosis.

## 3. Results

### 3.1. Mechanical and Optical Parameters Results

The YM values for all the tested variants were close to 2 MPa, which is comparable to the Young’s modulus measured for healthy tissues [30]. Similar values were reported for cylindrical scaffolds fabricated using other elastic polymers. For example, blow-spun vascular prostheses fabricated from polyurethanes with a hardness of 75A YM = 2.5 MPa [34] were obtained.

**Figure 5:**
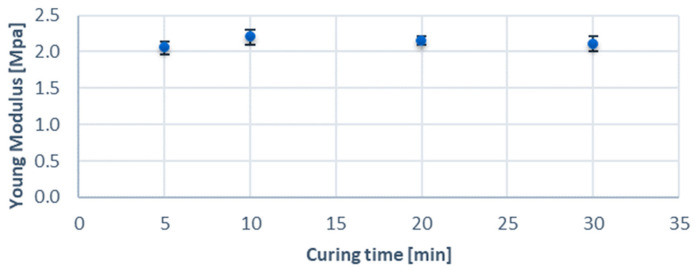
Young’s modulus [MPa] vs. curing time.

For a given geometry, six variants differing in curing time were analyzed (5, 10, 20, and 30 min). The results showed that the curing time did not affect the Young’s modulus values, as shown in Figure 5. ANOVA analysis with Tukey’s post hoc test showed that the differences between all variants were not statistically significant (*p* < 0.05). The mean density and refractive index were 1059 ± 22 kg/m^3^ and 1.473 ± 0.002, respectively. The density of the printed material was close to that of healthy tissue, which, combined with a similar Young’s modulus, makes it possible to create models with mechanical properties similar to those of the arteries. In addition, the refractive index was lower than that of the Clear V4 resin from (Formlabs, Somerville, MA, USA) [35], which allows for more accessible µPIV measurements using solutions with a lower NaI concentration or with NaSCN, which translates into a reduction in measurement costs.

The print quality was assessed by comparing the dimensions of the desired model with those of the printed model. The diameter dimensions of the printed system in Figure 2A were 5.01 ± 0.04 mm and 0.97 ± 0.04 mm compared to 5.00 and 1.00 mm for the 3D model. The stenosis dimensions from Figure 3d were also compared, obtaining 2.25 ± 0.04 mm compared to the expected 2.20 mm. The obtained print accuracy was similar to that of a print using Clear V4 resin from Formlabs (Somerville, MA, USA) [35], which does not have elastic properties.

### 3.2. Percutaneous Carotid Artery Stenting on 3D-Printed Phantoms

**Figure 6:**
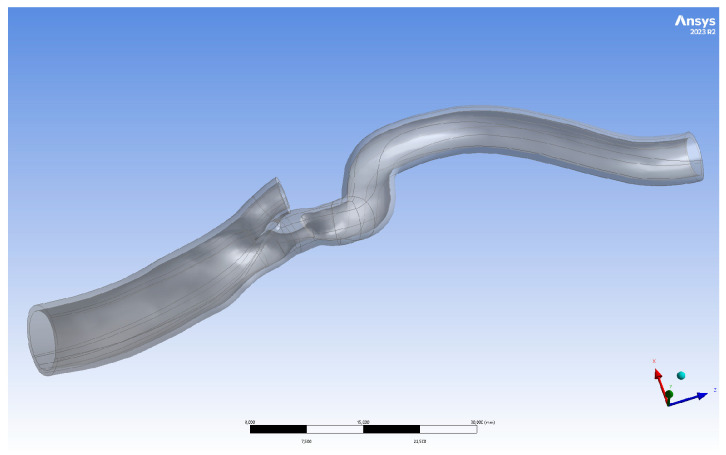
Three-dimensionally printed geometry I after post-processing.

The geometry of the artery in an 81-year-old male with 50% symptomatic right carotid artery stenosis is shown in Figure 6. A 9 × 40 mm carotid WALLSTENT™ catheter was implanted to restore the nominal lumen of the vessel.

**Figure 7:**
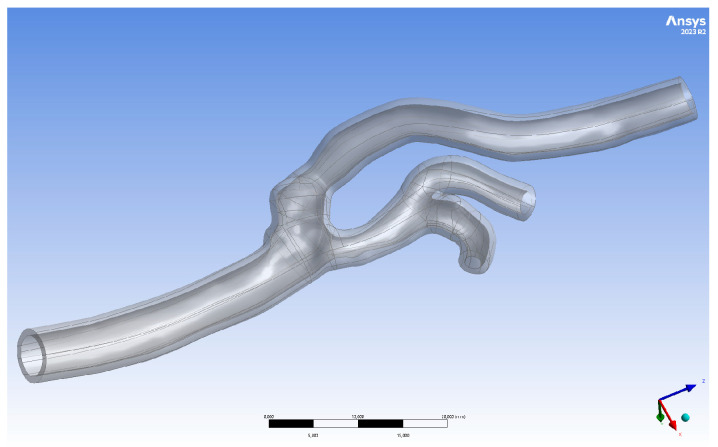
Three-dimensionally printed geometry II after post-processing.

The geometry of the artery in a 70-year-old male with 40% right carotid artery stenosis is shown in Figure 7. A 9 × 40 mm carotid WALLSTENT™ catheter was implanted to restore the nominal lumen of the vessel.

**Figure 8:**
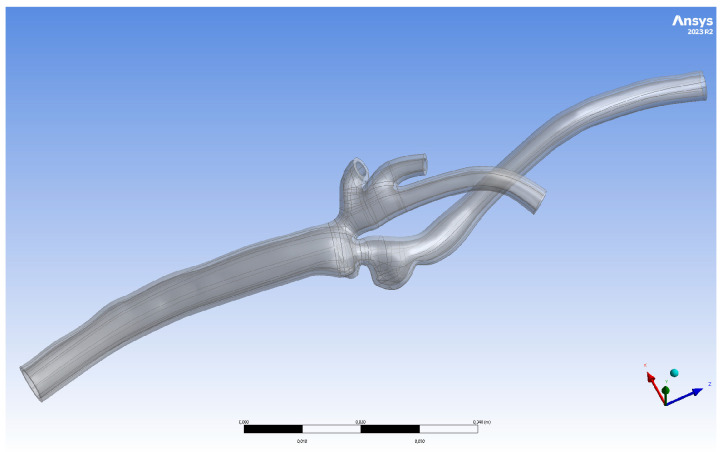
Three-dimensionally printed geometry III after post-processing.

The geometry of the artery of a 78-year-old male with 50% multiple levels of symptomatic left carotid stenosis and deep plaque ulceration is presented in Figure 8. A 9 × 40 mm carotid WALLSTENT™ was implanted to restore the nominal lumen of the vessel and cover the ulcer. 

**Figure 9:**
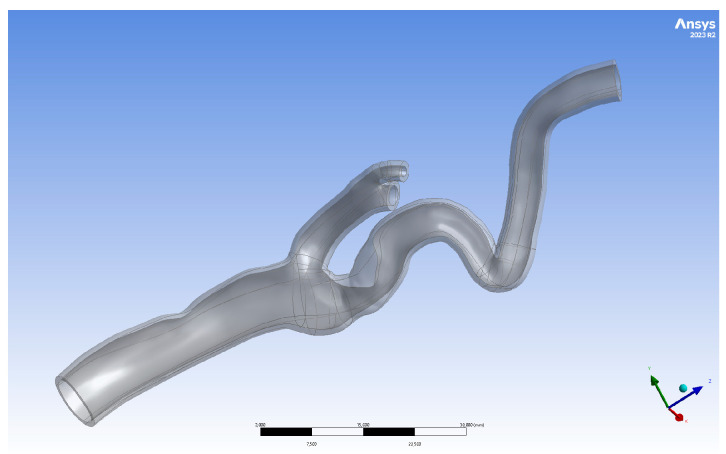
Three-dimensionally printed geometry IV after post-processing.

The geometry of the artery in a 77-year-old female with 70% symptomatic right carotid artery stenosis is presented in Figure 9. A tapered 6/8 × 40 mm Protégé™ RX carotid stent was implanted to restore the nominal lumen of the vessel. 

**Figure 10:**
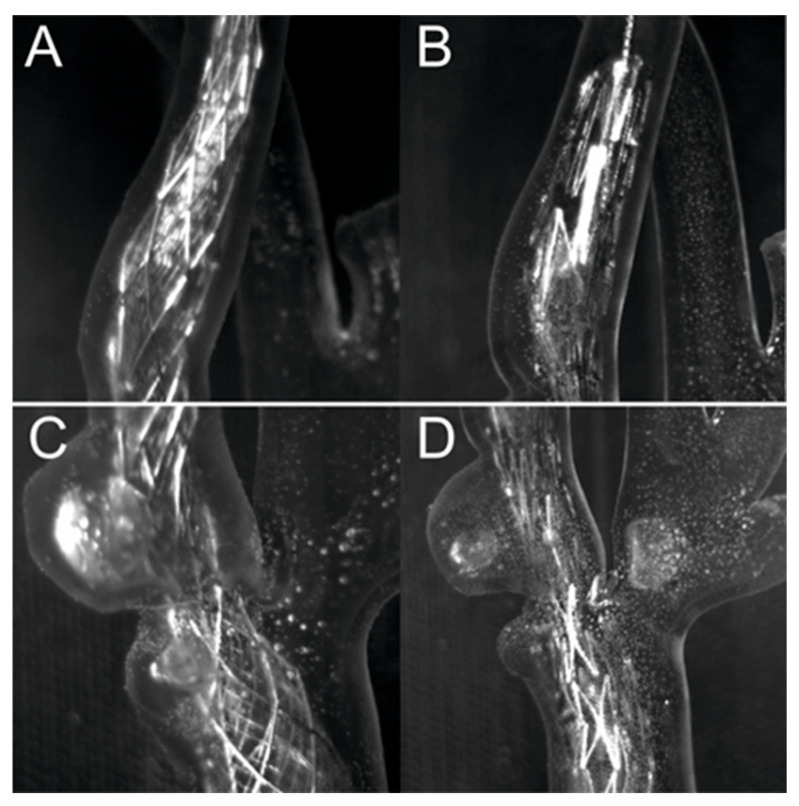
Comparison of stents for two exposures, (**A**,**C**)—dense mesh (Roadsaver™—Carotid Artery Stent), (**B**,**D**)—sparse mesh (Protégé™ RX—Carotid Artery Stent).

The artery models presented in Figure 8 and Figure 9 were used to assess the print quality using a solution containing rhodamine B (Figure 3b–d). The artery model shown in Figure 8 was used to compare the Roadsaver™ and Protégé™ RX stents. The deformation of the severe atherosclerotic stenosis is presented in Figure 10 using two copies of the model shown in Figure 8. Moreover, to demonstrate the deformation of the Carotid WALLSTENT™ during balloon inflation, the stent and model shown in Figure 7 were used, as shown in Figure 12. Detailed information on the stents used is presented in Table 1.

Figure 10 shows a comparison between the Roadsaver™ and Protégé™ RX stents. Figure 10 shows two exposures for two different stent models; the first close-up shows the distal fragment of the internal carotid artery, and the second shows the fragment with the ulceration and narrowing immediately distal to the branching of the carotid artery into the internal and external carotid artery. Comparing Figure 10A,C with Figure 10B,D, it can be seen that the Protégé™ RX stent with open-cell design adapts better to the artery model wall than the Roadsaver™ stent with a mesh design.

**Figure 11:**
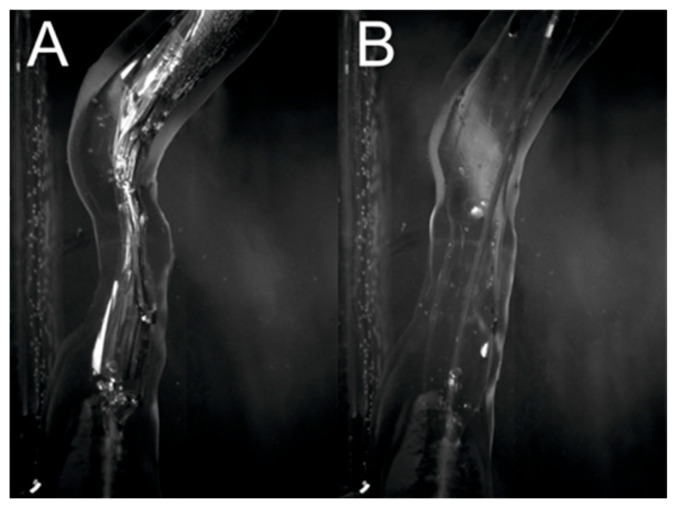
Comparison of artery model before (**A**) and after (**B**) inflating the balloon.

Figure 11 compares the artery models before and after balloon inflation. The measured inner diameter of the model was 2.13 mm before inflating the balloon and 4.05 mm after. The obtained dilation allowed the full lumen of the vessel with the inflated balloon to be reached. In addition, the vessel straightened as the balloon inflated, following the behavior of the vessel during an actual procedure. The obtained results prove that the printouts allow for high-quality pretreatment training by simulating the elastic properties of arteries, which is a valuable tool for doctors to reduce the risks associated with arterial angioplasty procedures in patients with atherosclerosis.

**Figure 12:**
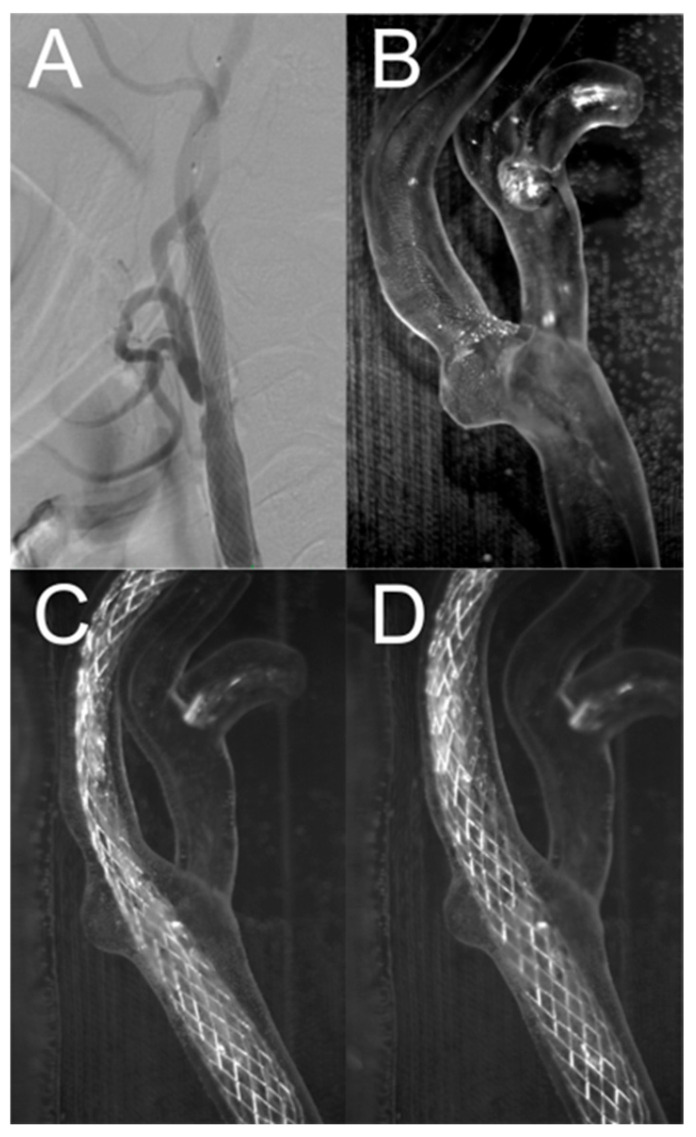
Comparison of artery with Carotid WALLSTENT™ after angioplasty with stent placement (**A**) and carotid artery model (**B**) with carotid artery stent before (**C**) and after (**D**) inflating the balloon.

Figure 12A shows a close-up of the CT angiography image of the inserted stent after percutaneous angioplasty. Figure 12B shows a close-up of the artery geometry without a stent. Figure 12C,D show the artery models before and after balloon inflation, respectively. As shown in Figure 12C, the deployed stent did not produce sufficient radial force to restore the nominal lumen of the vessel. After balloon inflation, the vessel lumen was restored, with the stent closely adhering to the model wall, and the artery model visibly straightened, as shown in Figure 12D. The changes in the shape of the vessel and stent were similar to those observed after percutaneous angioplasty performed on a living patient, as shown in Figure 12A.

**Figure 13:**
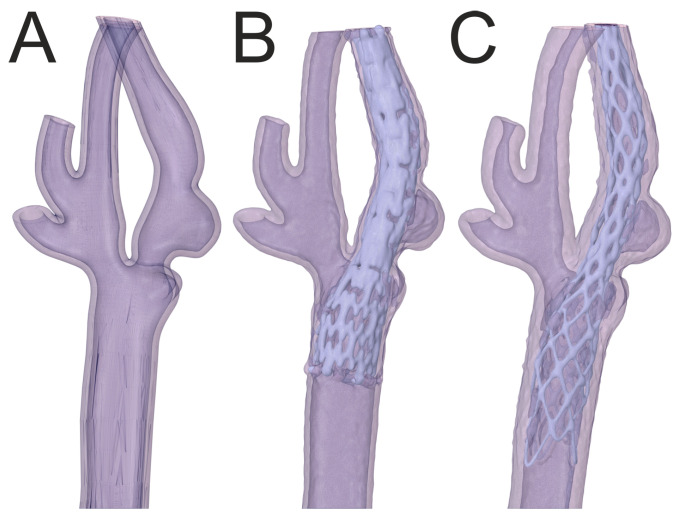
Comparison of CT images: (**A**) geometry without stent, (**B**) with Roadsaver™—Carotid Artery Stent, (**C**) with Protégé™ RX—Carotid Artery Stent.

Figure 13 shows that tomographic examination without contrast allowed us to obtain a good image of the geometry of the primary layer of the stent. The inner layer with a finer mesh is barely visible on cone-beam CT—the detail is lost in tomographic volumetric reconstruction using open-source In Vesalius 3.1. Comparing the fit of stents to the geometry of the vessel, it can be seen that a stent with an open-cell design better adapts its shape to the geometry of the artery and adheres better to the walls. The obtained results were entirely consistent with the observations obtained using the camera.

**Figure 14:**
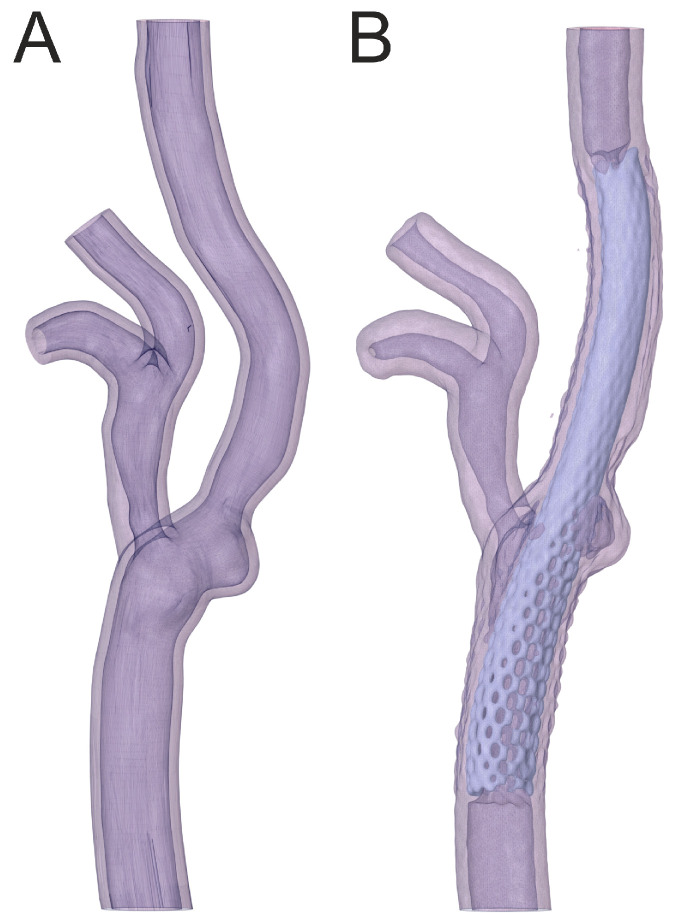
Comparison of CT images: (**A**) geometry without stent, (**B**) with Carotid WALLSTENT™.

Figure 14 compares the pre- and post-stent geometries using non-contrast cone-beam CT imaging. Comparing the geometries, it can be seen that the introduction of the stent led to the straightening of the arterial branch. Notably, the stent adheres to the vessel wall, as in the case of the camera images 

## 4. Discussion

The development of 3D printing contributes to its increasing use in medicine [36]. Three-dimensional printing methods such as Material Jetting (PolyJet) [37], stereolithography (SLA) [35,38], Fused Deposition Modeling (FDM) [38], Powder Bed Fusion (PBF) [39], or Binder Jetting [40,41] are helpful in medical practice. SLA printing and Material Jetting (PolyJet) are widely used in medical practice. The main reason is their high print resolution and relatively low equipment costs. The advantages of SLA printing are its high print resolution, relatively low equipment costs, and simplicity of use. 

Three-dimensionally printed models are the next step in incorporating anatomical, physiological, and functional changes through the cardiac cycle—the success of every procedure amounts to proper device selection and deployment. No 3D-printed models exist to explain the pitfalls during the stent deployment or angioplasty. Printed models emulated healthy tissue in terms of elasticity, which might curb their value in angioplasty-related scenarios; however, as shown in Figure 12, experimental conditions successfully matched real-life experience, both of stent placement and post-implantation angioplasty effect. An increasing number of patients is referred for carotid angioplasty, often with multiple comorbidities, with challenging plaque morphology, who would otherwise be undergoing endarterectomy. A 3D-printed model of a complex vascular channel created by the plaque would enable experimenting on the best hardware or maneuver to pass the lesion uneventfully, i.e., a distal protection device or microguidewire variant stiffness guidewires. 

Severe stenosis and the complex residual vascular channel of carotid disease shift the pathophysiology slightly towards flow-related pathology, as opposed to classic embolic disease. A 3D-printed elastic artery can be used to experimentally measure the constant Resistance Ratio (cRR) or Fractional Flow Ratio (FFR). FFR-guided revascularization is the standard of care for patients with coronary artery disease, and its value in carotid disease is to be established. Since FFR is a pressure wire-based index, the experimental setup would focus both on its successful placement in pivotal segments of the artery and on flow-related changes produced by the stenosis. Prospective studies on 3D-printed elastic models with variant FFR hardware could lead to better understanding of carotid disease and benefit new device development.

In order to systematize the printing parameters, a list of process and material parameters is summarized in Table 2.

## 5. Limitations

This study is retrospective in nature, which may lead to bias. Prospective studies using pre-procedural planning with the model and comparing the results with actual results would be more valuable. The main limitation of SLA technology is the possibility of 3D printing only from one material at a time, unlike Material Jetting (PolyJet); however, higher equipment and maintenance costs must be taken into account in the case of the second technology. An appropriate model of variable elasticity, reflecting the mechanical behavior of the carotid atherosclerotic plaque, requires the use of technology that allows for multi-material 3D printing. Unfortunately, this technology is more expensive compared to conventional SLA printing, which is associated with higher financial outlays and longer image pre-processing time and requires higher resolution medical imaging to distinguish between atherosclerotic plaque, calcifications, and healthy fragments of the artery wall. Further research is considering Material Jetting (PolyJet) technology, which allows for simultaneous printing of multiple materials with quality comparable to SLA printing.

Due to the limited availability of models and patients with cholesterol stenoses other than those of the carotid arteries, the study was limited to patients of the Military Institute of Medicine diagnosed and undergoing carotid angioplasty procedures. In the future, expanding the patient pool will be considered in cooperation with other medical centers. At the present stage, one should regard the presented 3D-printed models as an experimental setup, and their value as clinical training tools would be evaluated upon their further development and validation, together with the incorporation of other segments of the vascular tree.

## 6. Conclusions

The developed methodology for preparing and printing 3D blood vessel models allowed us to obtain personalized blood vessel phantoms within 1 day. The material used had mechanical properties similar to those of blood vessels, allowing the deformation of blood vessels to be considered when planning complex procedures. The material used was certified as safe for use. The refractive index of the material is very close to that of pure glycerin, which allows the treatment to be practiced in a system immersed in glycerin, which is non-toxic and inexpensive. An appropriate refractive index and high transparency allow training using an inexpensive optical system without expensive imaging methods, including radiation. The presented methodology allows individual workshop stations for angioplasty training with stent implantation using safe and affordable parts. Moreover, it was demonstrated that it is possible to compare the behaviors of different stents for a given geometry to select the appropriate stent. In summary, the developed methodology and system can improve the quality of education for interventional radiologists and may be a useful tool in the daily practice of clinicians in medical facilities. Patient education might also be improved using these models.

## Figures and Tables

**Table 1:** Summary of information on stents used.

Stent Type	Roadsaver™	Protégé™ RX	WALLSTENT™
Company	Terumo (Leuven, Belgium)	Medtronic (Plymouth, MA, USA)	Boston Scientific (Marlborough, MA, USA)
Type of the stent	Closed cell–braided	Open cell–laser cut	Closed cell–braided
Layers	Double layers	Monolayer	Monolayer
Free cell area [mm^2^]	0.0381	10.71	1.09
Material	Nitinol	Nitinol	Cobalt–chromium
Scaffolding (metal–artery ratio) [%]	39	29	15
Foreshortening [%]	27	8	49

**Table 2:** Summary of information for 3D-printing process and materials.

Parameter	Value
Resin type	Biomed Elastic 50A V1
Average print time	12 h
Average post-processing time	4 h
Material refractive index	1.473 ± 0.002 [-]
Young’s modulus	2.13 ± 0.05 MPa
Density of a 3D print	1059 ± 22 kg/m^3^

## Data Availability

Geometry files are provided as Supplementary Materials. Other data will be made available upon reasonable request.

## Supplementary Materials

The following supporting information can be downloaded at: https://www.mdpi.com/article/10.3390/jcm13175115/s1, zip folder of geometries in STL format.
